# Weight gain during the dolutegravir transition in the African Cohort Study

**DOI:** 10.1002/jia2.25899

**Published:** 2022-04-13

**Authors:** Allahna L. Esber, David Chang, Michael Iroezindu, Emmanuel Bahemana, Hannah Kibuuka, John Owuoth, Valentine Singoei, Jonah Maswai, Nicole F. Dear, Trevor A. Crowell, Christina S. Polyak, Julie A. Ake, the AFRICOS Study Group

**Affiliations:** ^1^ U.S. Military HIV Research Program Walter Reed Army Institute of Research Silver Spring Maryland USA; ^2^ Henry M. Jackson Foundation for the Advancement of Military Medicine Bethesda Maryland USA; ^3^ HJF Medical Research International Abuja Nigeria; ^4^ HJF Medical Research International Mbeya Tanzania; ^5^ Makerere University‐Walter Reed Project Kampala Uganda; ^6^ U.S. Army Medical Research Directorate – Africa Kisumu Kenya; ^7^ HJF Medical Research International Kisumu Kenya; ^8^ HJF Medical Research International Kericho Kenya

**Keywords:** Africa, antiretroviral therapy, body mass index, dolutegravir, HIV integrase inhibitors, weight gain

## Abstract

**Introduction:**

Dolutegravir (DTG) has become a preferred component of first‐line antiretroviral therapy (ART) in many settings but may be associated with excess weight gain. We evaluated changes in weight and body mass index (BMI) after switch to single‐tablet tenofovir/lamivudine/dolutegravir (TLD) by people living with HIV (PLWH) in four African countries.

**Methods:**

The African Cohort Study (AFRICOS) prospectively follows adults with and without HIV in Kenya, Uganda, Tanzania and Nigeria. Demographics, ART regimen, weight, BMI and waist‐to‐hip ratio were collected every 6 months. Multivariable Cox proportional hazards modelling was used to estimate hazard ratios and 95% confidence intervals (CIs) for factors associated with developing a BMI ≥25 kg/m^2^. Linear mixed effects models with random effects were used to examine the average change in BMI, weight and waist‐to‐hip ratio.

**Results:**

From 23 January 2013 to 1 December 2020, 2950 PLWH were enrolled in AFRICOS and 1474 transitioned to TLD. In adjusted models, PLWH on TLD had 1.77 times the hazard of developing a high BMI (95% CI: 1.22–2.55) compared to PLWH on non‐TLD ART. Examining change in weight among all PLWH on ART, participants on TLD gained an average of 0.68 kg (95% CI: 0.32–1.04) more than PLWH on other regimens after adjusting for duration on ART, sex, age, study site and CD4 nadir. Among participants who switched to TLD, the average change in weight prior to TLD switch was 0.35 kg/year (95% CI: 0.25–0.46) and average change in weight was 1.46 kg/year (95% CI: 1.18–1.75) in the year following transition to TLD after adjustment for confounders.

**Conclusions:**

Elevated BMI and weight gain among PLWH on TLD are concerning safety signals. Implications for the development of metabolic comorbidities should be monitored, particularly if annual weight gain persists during continued follow‐up after transitioning to TLD.

## 1 INTRODUCTION

Advancements in antiretroviral therapy (ART) have increased health and life expectancy among people living with HIV (PLWH) [1]. However, PLWH are now facing increasing morbidity and mortality due to non‐communicable diseases (NCDs) [2]. The risk of development of many NCDs, such as cardiovascular disease and diabetes, is closely associated with weight [3]. Among PLWH, weight gain is associated with even higher risk of developing NCDs as compared to individuals without HIV [4, 5]. Weight gain after ART initiation is often expected as a return‐to‐health phenomenon due to increased catabolism and decreased HIV‐associated inflammation [6]. However, weight gain has also been observed after ART switches to regimens containing integrase strand transfer inhibitors (INSTIs) in otherwise healthy PLWH with a long history of suppressive ART use [7].

Dolutegravir (DTG) is an INSTI that has become a popular anchor for ART regimens because it has a high barrier to resistance, is dosed once daily and does not require pharmacologic boosting [8]. This favourable profile led to endorsement by the World Health Organization (WHO) in 2018 as a preferred first‐line agent for the treatment of HIV [9]. The U.S. President's Emergency Plan for AIDS Relief (PEPFAR) and country partners began transitioning patients in supported HIV care programs to a single‐tablet regimen of tenofovir/lamivudine/dolutegravir (TLD) in late 2018 [10]. DTG usage continues to grow, particularly in low‐ to middle‐income countries [11, 12].

With the expansion of DTG, it is important to characterize potential deleterious effects among PLWH. Of all the INSTIs, weight gain is most prominent with second‐generation INSTIs, such as DTG [13]. Weight gain with DTG has predominantly been described in developed countries, ranging from 0.63 to 4.07 kg [7, 14, 15], and in ART‐naïve PLWH [16, 17]. This study adds to the limited pool of data for evaluating the potential for weight gain associated with DTG use in low‐ to middle‐income countries with a focus on switching to TLD during the PEPFAR programmatic rollout in four African countries.

## 2 METHODS

### 2.1 Study setting and population

The African Cohort Study (AFRICOS) is an ongoing prospective cohort that began enrolling adults and adolescents engaged in care at 12 PEPFAR‐supported clinics at five study sites in Kayunga, Uganda; South Rift Valley, Kenya; Kisumu West, Kenya; Mbeya, Tanzania; and Lagos and Abuja, Nigeria in January 2013 [18]. Study visits occur every 6 months. PLWH were recruited from randomized lists of current clinic clients and new diagnoses. Participants living without HIV (PLWoH) were recruited from community members accessing HIV testing services at the enrolling PEPFAR clinic. A small subset of participants were recruited from prior research studies conducted at the sites. Inclusion criteria included all adults ≥ 18 years of age, intention to be a long‐term resident of the area, willingness to provide contact information, consent to data and specimen collection and storage for future use, and ability to understand English or local language. Beginning in January 2020, enrolment was expanded to individuals aged ≥ 15 years.

The study was approved by institutional review boards of the Walter Reed Army Institute of Research, Makerere University School of Public Health, Kenya Medical Research Institute, Tanzania National Institute of Medical Research and Nigerian Ministry of Defense. All participants provided written informed consent.

### 2.2 Outcomes

Participants provided a medical history, completed a physical examination and underwent laboratory assessments every 6 months. Weight was recorded in kilograms (kg), and height was measured in centimetres (cm) at each visit. Body mass index (BMI) was calculated from these parameters. Waist and hip circumference were measured in cm at each study visit and used to calculate the waist‐to‐hip ratio (waist circumference divided by hip circumference). Study staff at all sites were trained in waist and hip measurement techniques, but location and phase of respiratory breath for the measurement were not standardized.

### 2.3 Exposures

PLWH underwent confirmatory HIV rapid diagnostic testing at the enrolment visit and PLWoH completed an HIV rapid diagnostic test at each visit. Study clinicians performed medical record review and extracted ART start date and regimen at every visit. Viral load testing was performed at each visit for PLWH using nucleic acid amplification methods [19]. CD4 T cell count was assessed at every visit for PLWH.

Demographics, including date of birth and sex, were captured at the enrolment visit. Age was left as a continuous variable with fitted splines. All data were recorded on paper case report forms and double entered into the ClinPlus platform (DZS Software Solutions, Bound Brock, NJ).

### 2.4 Analyses

Pearson chi‐squared tests were used to compare demographics and other enrolment characteristics among participants with and without HIV and, for PLWH, among those who switched to TLD and those who did not switch. Loess curves were fit to the raw data for BMI, weight and waist‐to‐hip ratio stratified by study site, sex, age and previous regimen.

In order to distinguish standard weight gain, return to health weight gain and weight gain associated with TLD use, we used three different analytic populations to compare changes in BMI and weight: all participants (PLWH and PLWoH), all PLWH on ART and only PLWH who transitioned to TLD. For all three populations, participants without any height measurement over the course of follow‐up were excluded. Additionally, visits where a participant recorded an ongoing pregnancy were excluded although these participants were eligible for inclusion at non‐pregnant visits. We used complete‐case analysis by visit. If participants had at least one visit with recorded height, sex or date of birth, the measurement was imputed to any visit with missing data. For variables such as weight that may fluctuate between visits, we did not impute values. Incidence rates and corresponding 95% confidence intervals (CI) of developing a high BMI (≥25 kg/m^2^) were calculated for all participants with a BMI at enrolment of ≤25 kg/m^2^ using total person‐time at risk for the following categories: PLWH on a non‐TLD regimen, ART‐naïve PLWH, PLWH on TLD and PLWoH. This variable was time‐varying, thus, a participant could contribute time to multiple categories. Cox proportional hazard modelling was used to estimate unadjusted and adjusted hazard ratios (HRs) for time to high BMI comparing those who switched to TLD with those on non‐integrase inhibitor regimens, were ART naïve or PLWoH. Enrolment into AFRICOS was baseline for the survival analysis. Only participants who did not have a BMI≥25 kg/m^2^ at the enrolment visit were included in this analysis. Participants who were lost to follow‐up or died during follow‐up were censored at their last study visit. ART status was a time‐varying covariate. Models were adjusted for study site, sex, age and BMI at enrolment. The proportionality of hazards assumption was assessed using Schoenfeld residuals.

We then restricted our analytic population to only PLWH who had at least one visit on ART during the study period. Linear mixed effects models with each participant treated as a random intercept were used to examine trends over time in BMI, weight and waist‐to‐hip ratios comparing participants who switched to TLD with those who remained on non‐TLD regimens. Baseline for these analyses was ART initiation. Spline terms were included to allow for different slopes in the first 6 months after ART initiation and greater than 6 months after ART initiation. Models were adjusted for age, sex, CD4 nadir, duration on ART and study site.

Last, we restricted our analyses to only participants who transitioned to TLD. Participants with weight and ART data at the visit before and after TLD switch were included in these analyses. To estimate the average changes in BMI, weight and waist‐to‐hip ratio over time, we used linear mixed effects spline models with knots at the time of switch and 1‐year post‐TLD switch. Models were adjusted for age, sex, CD4 nadir, prior ART regimen and study site.

### 2.5 Sensitivity analyses

To better characterize the changes in weight and BMI in excess of return‐to‐health among those who switched to TLD, we performed a sensitivity analysis restricting to participants with HIV RNA <1000 copies/ml and CD4 >500 cells/mm^3^ at the time of TLD switch [20].

To assess the influence of participants who were lost to follow up early in the study, we repeated analyses restricting to participants with at least one visit after 1 January 2019.

Analyses were performed in SAS 9.3 (SAS, Cary, NC) and Stata 16.0 (StataCorp, College Station, TX).

## 3 RESULTS

As of 1 December 2020, 3556 participants were enrolled in AFRICOS, including 2950 PLWH. Three participants were missing height data and 956 visits were missing weight (3.4% of total visits). The total number of person‐years of observation was 55,520 with 1332 person‐years of observation on TLD. There were 1433 participants who switched to TLD, and the median time on TLD was 1.03 years (interquartile range [IQR]: 0.76–1.35). Other INSTIs, such as raltegravir and elvitegravir, were not used by any AFRICOS participants during the observation period.

Participants had a median age at enrolment of 38.0 (IQR: 30.8–45.8) years and 2056 (57.8%) were females. All participants were ≥ 18 years. Among PLWH on ART, participants who switched to TLD were more likely to be older (median age 41.5, IQR: 28.9–43.0 vs. median age 35.6, IQR: 29.2–42.7; *p*<0.001), male (52.2% vs. 30.6%; *p*<0.001) and seeking care at the Kisumu West, Kenya site compared to those who did not switch (Table 1). At the time of TLD transition, the median age was 45.5 years (IQR: 38.8–53.0), the median BMI was 22.5 kg/m^2^ (IQR: 20.2–25.5 kg/m^2^) and the median weight was 62.6 kg (IQR: 55.5–70.0 kg). BMI varied significantly by sex at the time of TLD switch with 43.1% of females and 16.5% of males having a BMI ≥25 kg/m^2^ (*p*<0.001). Among the 50% of participants who gained weight 1 year after switching to TLD, 37% had a BMI ≥25 kg/m^2^ at the time of TLD switch. At the visit prior to switching to TLD, 73.9% were prescribed tenofovir/lamivudine/efavirenz and 23.1% were prescribed zidovudine/lamivudine/nevirapine. Less than 1% of participants were taking a protease inhibitor regimen prior to switching (*n* = 13) and 2.1% were on another regimen (*n* = 31).

**Table 1:** Participant characteristics at the enrolment visit by TLD switch status

	Participants without HIV	Did not switch to TLD	Switched to TLD	
	*N* = 606	*N* = 1485	*N* = 1433	*p*‐Value
Study site				**<0.001**
Kayunga, Uganda	105 (17.3%)	332 (22.4%)	197 (13.7%)	
South Rift Valley, Kenya	207 (34.2%)	584 (39.4%)	432 (30.1%)	
Kisumu West, Kenya	127 (21.0%)	178 (12.0%)	328 (22.9%)	
Mbeya, Tanzania	97 (16.0%)	265 (17.9%)	299 (20.9%)	
Abuja and Lagos Nigeria	70 (11.6%)	125 (8.4%)	177 (12.4%)	
Age (years)				**<0.001**
18–29	179 (29.6%)	359 (24.2%)	147 (10.3%)	
30–39	207 (34.2%)	573 (38.6%)	435 (30.4%)	
40–49	141 (23.3%)	387 (26.1%)	538 (37.5%)	
50+	78 (12.9%)	165 (11.1%)	313 (21.8%)	
Sex				**<0.001**
Male	265 (43.7%)	454 (30.6%)	748 (52.2%)	
Female	341 (56.3%)	1030 (69.4%)	685 (47.8%)	
Weight (kg)				**<0.001**
<56	121 (20.0%)	528 (35.6%)	417 (29.1%)	
56–62	151 (24.9%)	376 (25.4%)	351 (24.5%)	
62–71	156 (25.7%)	319 (21.5%)	382 (26.7%)	
>71	178 (29.4%)	260 (17.5%)	282 (19.7%)	
BMI (kg/m^2^)				**<0.001**
<18.5	38 (6.3%)	159 (10.7%)	159 (11.1%)	
18.5–24	347 (57.3%)	946 (63.7%)	901 (62.9%)	
≥25	221 (36.5%)	380 (25.6%)	373 (26.0%)	
Depressed				**0.029**
No	506 (83.6%)	1172 (79.1%)	1174 (81.9%)	
Yes	99 (16.4%)	310 (20.9%)	259 (18.1%)	
ART type				**<0.001**
Efavirenz	–	510 (34.3%)	646 (45.1%)	
Nevirapine		299 (20.1%)	391 (27.3%)	
Protease inhibitors		138 (9.3%)	8 (0.6%)	
Naive		188 (12.7%)	62 (4.3%)	
Other		350 (23.6%)	326 (22.7%)	
CD4 (cells/mm^3^)				0.073
<200	–	318 (21.7%)	250 (17.6%)	
200–349		359 (24.5%)	341 (24.0%)	
350–499		323 (22.0%)	317 (22.4%)	
500+		468 (31.9%)	510 (36.0%)	
Viral load (copies/ml)				**<0.001**
< 1000	–	791 (53.7%)	999 (70.2%)	
≥ 1000		681 (46.3%)	424 (29.8%)	
Time from ART initiation to TLD switch (years)			6.14 (4.34–9.74)	

Note: Data are *n* (%) for categorical variables and median (interquartile range) for continuous variables. Significant differences were assessed using Pearson chi‐squared tests. Bold indicates significance at *p*<0.05. Twenty‐three participants were missing viral load and 32 were missing CD4 measurements at the enrolment visit. Three participants were missing weight data at the enrolment visit.

Abbreviations: ART, antiretroviral therapy; TLD, tenofovir/lamivudine/dolutegravir.

### 3.1 All participants

Of the 2144 participants at risk, 516 participants developed a BMI ≥25 kg/m^2^ (Table 2). Unadjusted incidence rates of developing a high BMI were similar among participants without HIV and those on ART with the highest incidence among participants on non‐TLD ART (77.1 cases per 1000 person‐years [PY]; 95% CI: 69.8–85.2). After adjusting for sex, age at enrolment, study site and BMI at enrolment, PLWH on TLD had 1.77 times the hazard of developing a high BMI (95% CI: 1.22–2.55) compared to PLWH on non‐TLD ART, and ART‐naïve PLWH had the lowest rate (HR 0.43; 95% CI 0.27–0.70; Table 3).

**Table 2:** Unadjusted incidence rate of BMI ≥25 kg/m^2^

	Person‐time	Failures	Incidence rate	95% CI
PLWH on non‐TLD ART	5069.11	391	77.13	69.85–85.17
PLWH, ART naïve	503.77	19	37.71	24.06–59.13
PLWH on TLD	559.35	41	73.30	53.97–99.55
People without HIV	937.23	65	69.80	54.74–89.01

**Table 3:** Unadjusted and adjusted Cox proportional hazard analyses of BMI ≥25 kg/m^2^ (all participants)

	Unadjusted HR	95% CI	Adjusted HR	95% CI
HIV and ART status				
PLWH on non‐TLD ART	Ref		–	
PLWH, ART naïve	0.43	0.27–0.69	0.43	0.27–0.70
PLWH on TLD	1.40	0.98–2.02	1.77	1.22–2.55
People without HIV	0.91	0.70–1.18	0.89	0.68–1.17
Sex				
Male	Ref		–	
Female	2.57	2.13–3.10	2.24	1.85–2.73
Age at enrolment				
18–29	1.05	1.00–1.10	1.05	1.00–1.10
30–39	0.96	0.93–0.99	0.97	0.94–1.00
40–49	1.011	0.98–1.05	1.00	0.96–1.03
50+	0.95	0.90–0.99	0.98	0.94–1.03
Study site				
Uganda	Ref		–	
SRV, Kenya	1.84	1.41–2.39	1.45	1.11–1.89
Kisumu West, Kenya	1.14	0.84–1.54	0.96	0.70–1.30
Tanzania	3.18	2.35–4.30	1.93	1.41–2.65
Nigeria	3.00	2.10–4.29	1.63	1.13–2.34
BMI at enrolment (kg/m^2^)	1.81	1.71–1.91	1.73	1.64–1.83

Abbreviations: PLWH, people living with HIV; SRV, South Rift Valley; TLD, tenofovir/lamivudine/dolutegravir.

### 3.2 PLWH on ART

After adjusting for duration on ART, participants on TLD had an average change in BMI 0.40 kg/m^2^ greater than those who did not switch to TLD (95% CI: 0.27–0.53; Table 4). After adjusting for duration on ART, sex, age, study site and CD4 nadir participants who switched to TLD had a change in BMI 0.24 kg/m^2^ greater than those who did not switch (95% CI: 0.12–0.37).

**Table 4:** Unadjusted and adjusted average change in BMI before and after switching to TLD among all PLWH

	Unadjusted (kg/m^2^)	95% CI	Adjusted (kg/m^2^)	95% CI
On TLD				
No	Ref		–	
Yes	0.40	0.27–0.53	0.24	0.12–0.37
Time before ART initiation	−0.66	−0.84 to 0.48	−0.62	−0.80 to 0.44
<6 months after ART initiation	0.59	0.29–0.91	0.96	0.65–1.26
6+ months after ART initiation	0.02	−0.00 to 0.04	0.00	−0.02 to 0.02
Sex				
Male	Ref		−	
Female	2.31	2.19–2.42	2.36	2.24–2.48
Age at visit				
18–29	0.16	0.13–0.19	0.19	0.16–0.22
30–39	0.05	0.03–0.07	0.08	0.06–0.10
40–49	−0.01	−0.03 to 0.01	0.01	−0.01 to 0.03
50+	−0.06	−0.08 to 0.04	−0.03	−0.05 to 0.01
Study site				
Uganda	Ref		−	
SRV, Kenya	1.10	0.94–1.27	1.07	0.91–1.24
Kisumu West, Kenya	−0.29	−0.47 to 0.10	−0.18	−0.37 to 0.01
Tanzania	2.20	2.01–2.39	2.56	2.37–2.75
Nigeria	2.92	2.70–3.15	2.94	2.71–3.17
CD4 nadir				
<200	Ref		−	
200–349	0.31	0.17–0.45	0.52	0.39–0.66
350–499	0.12	−0.05 to 0.30	0.60	0.42–0.78
500+	0.78	0.59–0.97	1.23	1.03–1.43

Abbreviations: PLWH, people living with HIV; SRV, South Rift Valley; TLD, tenofovir/lamivudine/dolutegravir.

Among PLWH on ART, those who switched to TLD gained an average of 0.44 kg more than those who did not switch after adjusting for duration on ART (95% CI: 0.08–0.80; Table 5). After adjusting for sex, age at visit, study site and CD4 nadir, participants who switched to TLD gained on average 0.68 kg more than participants who did not switch to TLD (95% CI: 0.32–1.04).

**Table 5:** Unadjusted and adjusted average change in weight before and after switching to TLD among all PLWH

	Unadjusted (kg)	95% CI	Adjusted (kg)	95% CI
On TLD				
No	Ref		−	
Yes	0.44	0.08–0.80	0.68	0.32–1.04
Time before ART initiation	−2.16	−2.67 to 1.66	−2.55	−3.06 to 2.05
<6 months after ART initiation	2.43	1.58–3.30	2.45	1.60–3.30
6+ months after ART initiation	0.05	−0.00 to 0.11	−0.04	−0.10 to 0.02
Sex				
Male	Ref		−	
Female	−1.05	−1.39 to 0.72	−0.87	−1.21 to 0.54
Age at visit				
18–29	0.63	0.55–0.71	0.58	0.50–0.66
30–39	0.21	0.15–0.26	0.24	0.19 to 0.30
40–49	0.00	−0.05 to 0.06	0.02	−0.03 to 0.07
50+	−0.13	−0.19 to 0.07	−0.11	−0.17 to 0.05
Study site				
Uganda	Ref		−	
SRV, Kenya	4.95	4.50–5.39	4.98	4.52–5.45
Kisumu West, Kenya	1.90	1.38–2.41	2.07	1.53–2.60
Tanzania	4.30	3.78–4.83	5.53	4.99–6.07
Nigeria	10.05	9.42–10.68	10.21	9.56–10.85
CD4 nadir				
<200	Ref		−	
200–349	0.34	−0.05 to 0.73	1.21	0.82–1.60
350–499	−0.01	−0.50 to 0.47	1.54	1.04–2.04
500+	0.94	0.42–1.45	3.18	2.62–3.74

Abbreviations: SRV, South Rift Valley; TLD, tenofovir/lamivudine/dolutegravir.

Participants who switched to TLD had a mean increase in waist‐to‐hip ratio 0.01 (95% CI: 0.01–0.02) greater than those who did not switch to TLD after adjusting for duration on ART, sex, study site, age and CD4 nadir.

### 3.3 TLD switch only

**Figure 1:**
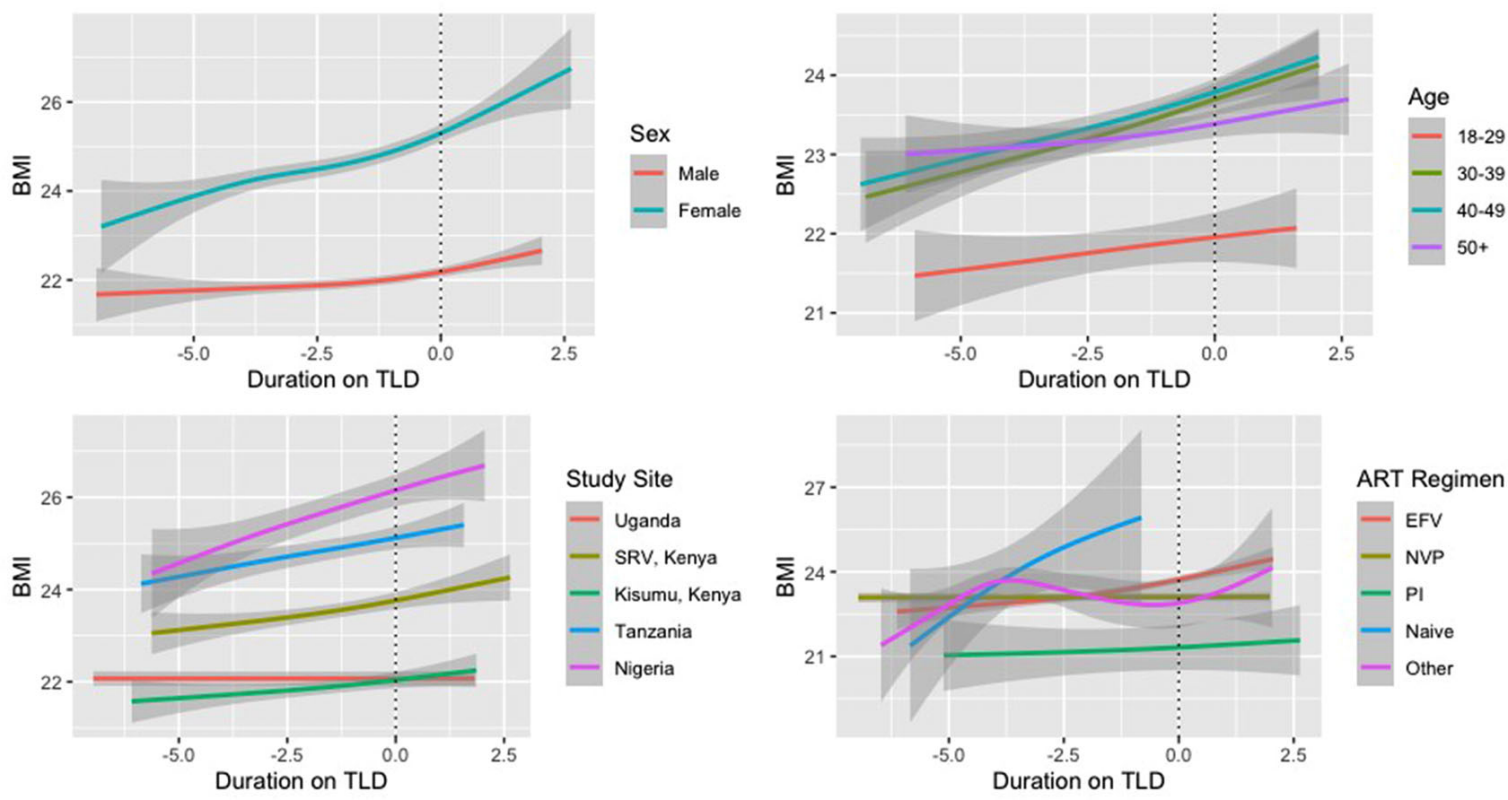
Average BMI (95% CI) by time since TLD switch and (a) sex, (b) age at visit, (c) study site and (d) previous regimen. Abbreviations: EFV, efavirenz‐based regimen; NVP, nevirapine‐based regimen; PI, protease inhibitor; SRV, South Rift Valley. Note: Shaded areas represent 95% confidence intervals. BMI measured in kg/m^2^ This figure presents average BMI over time by sex, age, study site and previous ART regimen among participants who transitioned to TLD.

Among PLWH who switched to TLD, there was a significant difference in the change in BMI between males and females in the first year after transitioning to TLD, but this did not differ significantly over time by age, study site or previous regimen (Figure 1). Among the participants who switched to TLD, average BMI increased by 0.13 kg/m^2^ per year (95% CI: 0.09–0.17) prior to TLD switch and 0.49 kg/m^2^ per year (95% CI: 0.37–0.60) in the first year after TLD switch with adjustment for sex, age, study site, CD4 nadir and prior ART regimen (Table 6). After 1 year on TLD, average annual change in BMI was 0.46 kg/m^2^ (95% CI: 0.07–0.86). Among participants who were virally suppressed and had a CD4 ≥ 500 at the time of TLD switch, there was an average change in BMI of 0.10 kg/m^2^ prior to TLD switch (95% CI: 0.04–0.16), average change in BMI within the year following TLD switch of 0.51 kg/m^2^ (95% CI: 0.34–0.69) and annual average change in BMI of 1.25 kg/m^2^ after a year on TLD (95% CI: 0.24–2.25). Findings were similar when restricting to participants with at least one visit after 1 January 2019.

**Table 6:** Unadjusted and adjusted average change in BMI before and after switching to TLD among participants who switch to TLD

	Unadjusted (kg/m^2^)	95% CI	Adjusted (kg/m^2^)	95% CI
Timing of visit relative to switch				
Time before TLD switch	0.11	0.06–0.15	0.13	0.09–0.17
<1 year after TLD switch	0.63	0.43–0.83	0.49	0.37–0.60
1+ year after TLD switch	0.16	−0.4 to 0.77	0.46	0.07–0.86
Sex				
Male	Ref		−	
Female	2.57	2.42–2.72	2.54	2.14–2.94
Age at visit				
18–29	0.19	0.14–0.24	0.16	0.07–0.25
30–39	0.04	0.01–0.07	0.07	0.02–0.11
40–49	−0.00	−0.03 to 0.02	0.04	0.01–0.08
50+	−0.04	−0.07 to 0.01	−0.05	−0.10 to 0.01
Study site				
Uganda	Ref		−	
SRV, Kenya	1.04	0.81–1.27	0.94	0.31–1.56
Kisumu West, Kenya	−0.34	−0.58 to 0.10	−0.54	−1.20 to 0.11
Tanzania	2.51	2.26–2.76	2.38	1.68–3.07
Nigeria	3.15	2.86–3.43	2.80	1.99–3.60
CD4 nadir				
<200	Ref		−	
200–349	0.23	0.05–0.41	0.53	0.21–0.84
350–499	−0.09	−0.32 to 0.14	0.84	0.46–1.22
500+	1.12	0.87–1.38	0.93	0.50–1.36
Prior regimen				
Efavirenz	Ref		−	
Nevirapine	−0.01	−0.21 to 0.20	0.48	−0.01 to 0.98
Other	−0.62	−1.18 to 0.06	−0.98	−2.25 to 0.28

Note: Interactions between time and sex, age, study site and prior regimen were assessed. There were no significant interactions with time in the year after TLD switch and sex, *p* = 0.85; age: 18–29, *p* = 0.55; 30–39, *p* = 0.60; 40–49, *p* = 0.96; 50+, *p* = 0.05; study site: Uganda, *p* = 0.41; SRV, Kenya, *p* = 0.77; Kisumu West, Kenya, *p* = 0.37; Tanzania, *p* = 0.57; Nigeria, *p* = 0.85; or prior regimen: efavirenz, *p* = 0.73; nevirapine, *p* = 0.54; other, *p* = 0.81.

Abbreviations: SRV, South Rift Valley; TLD, tenofovir/lamivudine/dolutegravir.

**Figure 2:**
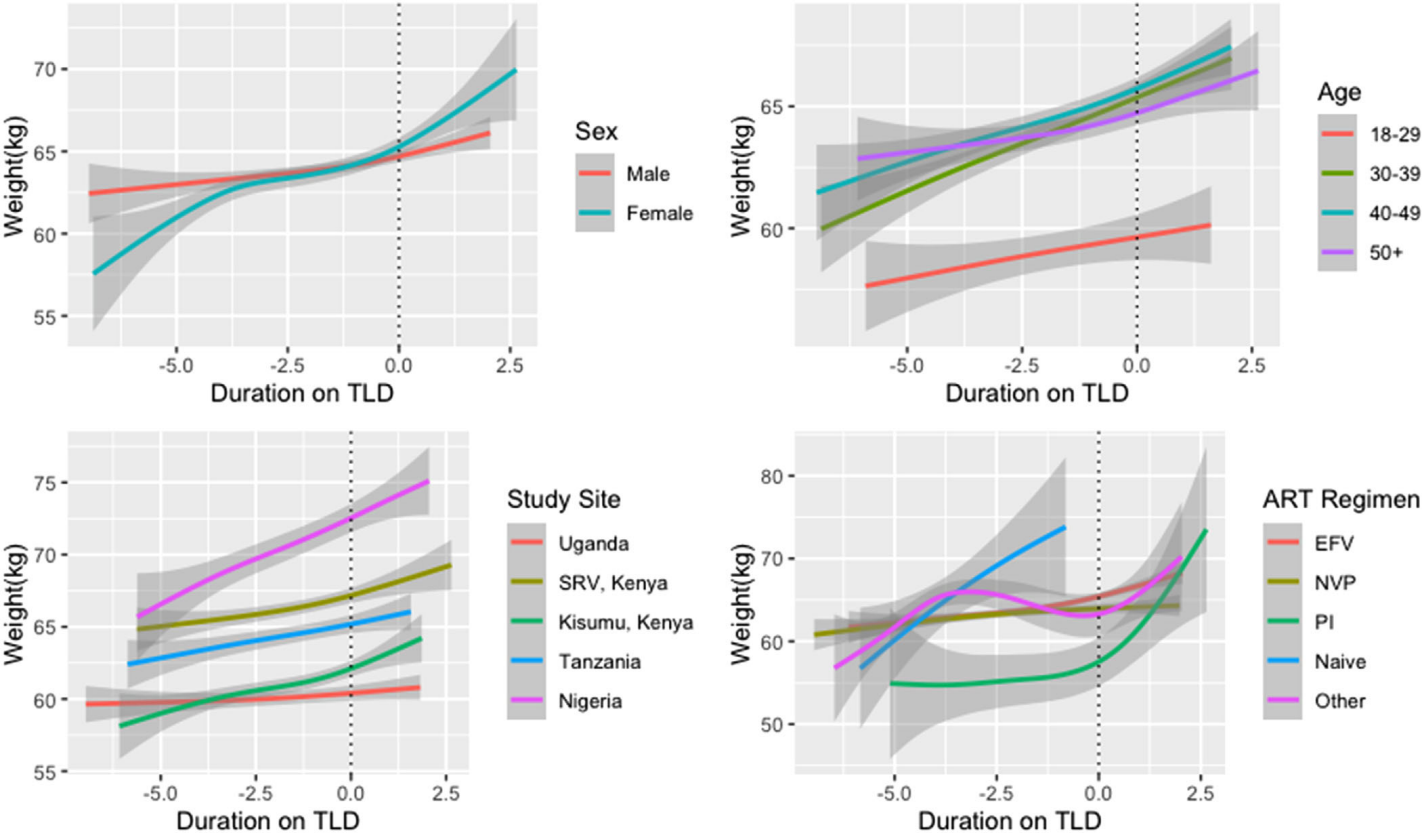
Average weight (95% CI) by time since TLD switch and (a) sex, (b) age at visit, (c) study site and (d) previous regimen. Abbreviations: EFV, efavirenz‐based regimen; NVP, nevirapine‐based regimen; PI, protease inhibitor; SRV, South Rift Valley. Note: Shaded areas represent 95% confidence intervals. This figure presents average weight over time by sex, age, study site and previous ART regimen among participants who transitioned to TLD.

Overall, we did not see significant differences in the interaction between time on TLD and age at visit, study site or previous regimen (Figure 2). Examining the interaction between duration on TLD and sex, females had a higher slope for weight gain compared to males although the difference in slope was no longer significant after a year on TLD. Of note, while we did not see any patterns by study site or age, participants seeking care at the Nigerian sites had higher average weight, while younger participants had a lower average weight compared to older participants throughout the period of follow up.

Among those who switched to TLD, the average adjusted change in weight prior to TLD switch was 0.35 kg/year (95% CI: 0.25–0.46) and average change in weight in the year following TLD transition was 1.46 kg/year (95% CI: 1.18–1.75; Table 7). After 1 year on TLD, the average annual change in weight was 1.36 kg (95% CI: 0.37–2.35). In the subset of virally suppressed participants, the average change in weight was 0.24 kg/year (95% CI: 0.09–0.39) prior to TLD switch, and 1.54 kg/year (95% CI: 1.11–1.98) in the year following TLD transition. Following the first year on TLD, average change in weight was 3.05 kg (95% CI: 0.56–5.53). Findings were similar to the main analysis when restricting to participants with at least one visit after 1 January 2019.

**Table 7:** Unadjusted and adjusted average change in weight before and after switching to TLD among participants who switch to TLD

	Unadjusted (kg)	95% CI	Adjusted (kg)	95% CI
Time before TLD switch	0.51	0.38–0.63	0.35	0.25–0.46
<1 year after TLD switch	1.54	1.00–2.08	1.46	1.18–1.75
1+ year after TLD switch	0.37	−1.30 to 2.04	1.36	0.37–2.35
Sex				
Male	Ref		−	
Female	−0.55	−0.98 to 0.13	−0.62	−1.79 to 0.55
Age at visit				
18–29	0.64	0.51–0.78	0.37	0.14–0.61
30–39	0.14	0.07–0.22	0.19	0.06–0.31
40–49	0.02	−0.04 to 0.09	0.14	0.03–0.24
50+	−0.12	−0.20 to 0.05	−0.17	−0.29 to 0.05
Study site				
Uganda	Ref		−	
SRV, Kenya	4.81	4.19–5.47	4.48	2.65–6.30
Kisumu West, Kenya	0.48	−0.18 to 1.14	0.78	−1.15 to 2.70
Tanzania	3.79	3.10–4.47	4.61	2.58–6.64
Nigeria	8.90	8.11–9.69	9.69	7.34–12.04
CD4 nadir				
<200	Ref		−	
200–349	0.10	−0.39 to 0.59	1.38	0.53–2.23
350–499	−0.05	−0.67 to 0.57	2.32	1.30–3.34
500+	2.50	1.81–3.20	2.78	1.61–3.94
Prior regimen				
Efavirenz	Ref		−	
Nevirapine	−0.22	−0.78 to 0.33	0.71	−0.73 to 2.15
Other	−2.68	−4.20 to 1.17	−3.41	−7.12 to 0.29

Note: Interactions between time and sex, age, study site and prior regimen were assessed. There were no significant interactions with time in the year after TLD switch and sex: male, *p* = 0.92; female *p* = 0.46; age: 18–29, *p* = 0.92; 30–39, *p* = 0.15; 40–49, *p* = 0.37; 50+, *p* = 0.11; study site: Uganda, *p* = 0.68; SRV, Kenya, *p* = 0.82; Kisumu West, Kenya, *p* = 0.07; Tanzania, *p* = 0.54; Nigeria, *p* = 0.61; or prior regimen: efavirenz, *p* = 0.71; nevirapine, *p* = 0.48; other, *p* = 0.45.

Abbreviations: SRV, South Rift Valley; TLD, tenofovir/lamivudine/dolutegravir.

**Figure 3:**
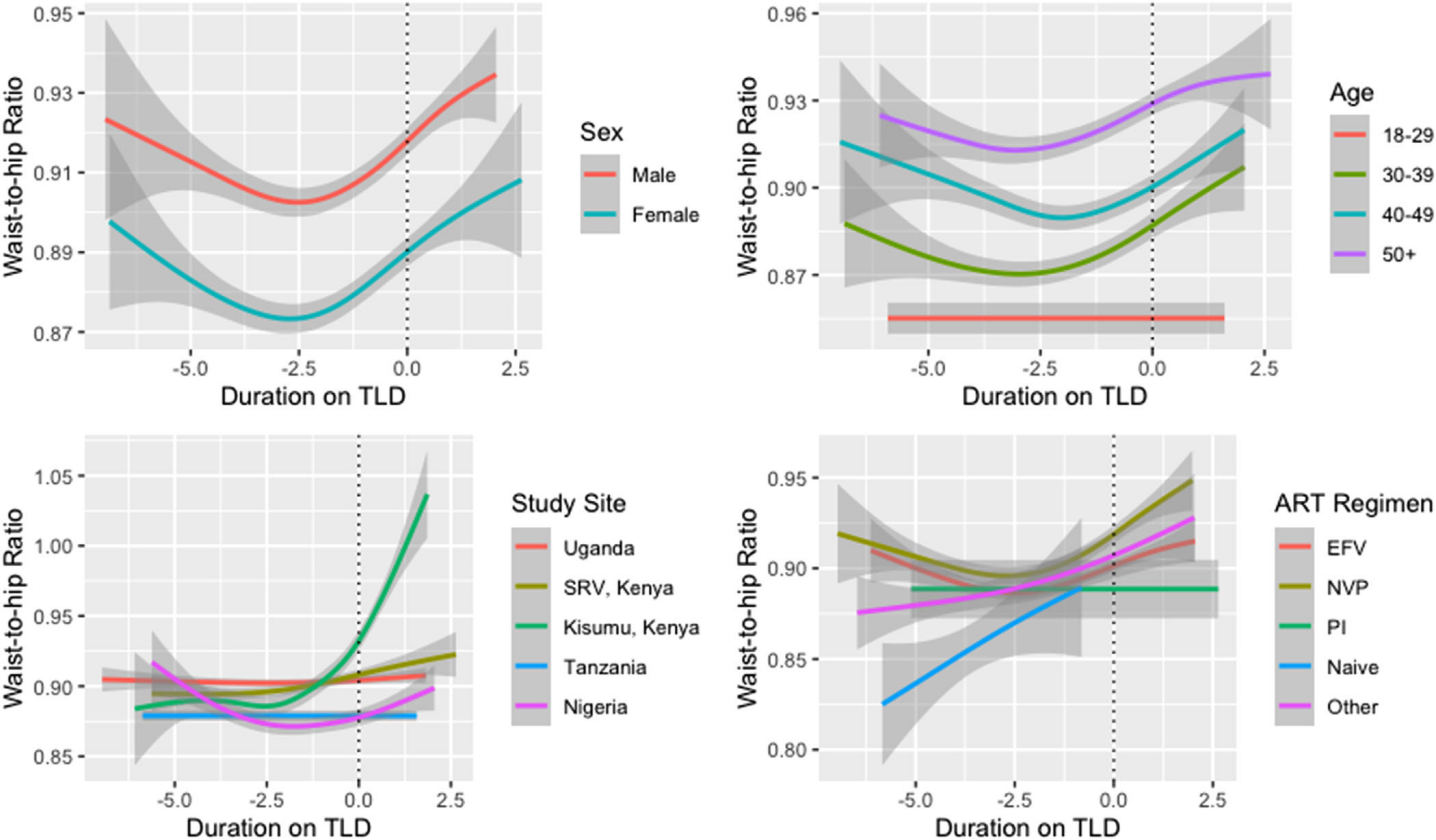
Average waist‐to‐hip ratio (95% CI) by time since TLD switch and (a) sex, (b) age at visit, (c) study site and (d) previous regimen. Abbreviations: EFV, efavirenz‐based regimen; NVP, nevirapine‐based regimen; PI, protease inhibitor; SRV, South Rift Valley. Note: Shaded areas represent 95% confidence intervals. This figure presents average waist‐to‐hip ratio over time by sex, age, study site and previous ART regimen among participants who transitioned to TLD.

We did not find a significant difference in waist‐to‐hip ratio over time by study site or previous regimen; however, changes in waist‐to‐hip ratio differed significantly within the first year of initiating TLD by sex and age (Figure 3). Among participants who switched to TLD, waist‐to‐hip ratio increased by an average of 0.02 (95% CI: 0.02–0.03) in the first year after TLD transition but did not differ significantly after 1 year on TLD.

## 4 DISCUSSION

Weight gain associated with DTG has been definitively described in treatment naïve PLWH initiating ART. In resource‐rich settings, newer INSTIs, particularly DTG, have been reported to have the highest weight gain in these ART naïve participants [7] with average weight gain ranging from 2.4 kg [21] up to 6 kg after 1 year on DTG [22]. A similar magnitude of weight gain has also been described in low‐ to‐middle‐income countries [16]. Weight gain upon switching to DTG is less well described. Among primarily virally suppressed, treatment‐experienced PLWH in sub‐Saharan Africa, we found a 1.3 kg weight gain and 0.44 kg/m^2^ increase in BMI among participants in the first year after transition to TLD. In AFRICOS, our weight gain findings are similar to other switch studies with between 1 and 3 kg of weight gain [13, 15]. In contrast, Burns et al. found minimal weight gain after transitioning to DTG or raltegravir and no change in the rate of weight gain pre and post switch among PLWH in the United Kingdom [14]. The differing findings may be due in part to different population characteristics, with the UK cohort having a higher BMI (25.3 vs. 22.7 kg/m^2^) and weight (76.6 vs. 63.0 kg) at the time of TLD switch. AFRICOS contributes to the growing body of literature demonstrating increased weight gain after TLD switch persisting past the first year of switching regimens.

A theorized aetiology for DTG‐related weight gain is the inhibition of the melanocortin‐4 receptor (MC4R). Inactivation of MC4R via knock outs results in obesity in mouse models [23], whereas agonists to MC4R in animal models and a small phase 1 human study induce weight loss [24]. DTG interaction with MC4R data is limited to in‐vitro data, where DTG inhibited alpha‐melanocyte‐stimulating hormone binding to human recombinant MC4R receptor [25]. The MC4R pathway has also been postulated to explain the differences in DTG weight gain by gender [15], but does not explain how some studies have seen greater weight gain switching from an non‐nucleoside reverse transcriptase inhibitors (NNRTI) pre‐switch regimen. An alternative mechanism that could account for the differences seen in pre‐switch regimens is by polymorphisms in efavirenz metabolism by CYP2B6. Slow metabolism of efavirenz by CYP2B6 polymorphisms has been found to be associated with increased weight gain switching from NNRTI to INSTIs, except for DTG [26]. Potentially, increased efavirenz levels lead to less pre‐switch weight gain through interference or augmentation of TDF appetite suppression. On a cellular level, INSTIs, such as DTG and elvitegravir, have been demonstrated to have negative effects on respiratory capacity in CD4^+^ T cells [27]. While this effect is likely to be broader than CD4^+^ T cells, its clinical relevance is unknown, particularly regarding metabolic comorbidities.

There have been prior studies demonstrating a more dramatic weight gain in women compared to men after switching to an INSTI regimen [15, 28]. In AFRICOS, there was also a difference in weight gain between men and women, with women having a greater weight gain 1 year post TLD switch, but this difference was not significant after the first year. Other factors, such as older age and prior regimens, have also been associated with increased weight gain after switching to an INSTI regimen [13, 15]. Regimens with an NNRTI anchor appeared to have greater weight gain rather than protease inhibitor (PI) anchors when transitioning to INSTI regimens in prior studies [15]. This association was not seen in AFRICOS. In contrast, prior regimens did not have a statistically significant difference in weight. The small sample of PI‐based pre‐switch regimens significantly limited our ability to analyse this question robustly.

Participants on TLD had higher rates of developing a high BMI, with a significant proportion having elevated BMI even prior to switch, particularly among women. TLD switches in AFRICOS resulted in a higher risk of overweight classification, but the clinical significance will need further investigation. We also observed a significant increase in waist‐to‐hip ratio after transitioning to TLD, and this was superimposed on a baseline median waist‐to‐hip ratio that already exceeded 0.90 for males and 0.85 for females, the WHO threshold value for abdominal obesity. Downstream effects, such as development or worsening of diabetes, hyperlipidaemia, hypertension and strokes that result in significant morbidity, will be important to investigate as low‐ to middle‐income countries increase the usage of DTG. These data will inform screening methodology and shape programmatic policy. Future studies should also evaluate the reversibility of weight gain.

Strengths of this study include the large and diverse participant population and extensive weight history prior to TLD switch, as well as up to 2 years of follow‐up time for participants after transitioning to TLD. However, the study is not without a few limitations. As the study was conducted at clinics where TLD was programmatically rolled out, those who switched to TLD were different than those who remained on older, non‐TLD regimens. To mitigate the differences in characteristics of participants who transitioned to TLD, we included analyses using the participants’ own weight trajectory prior to TLD switch. We were also unable to adjust for potential confounders of weight gain, such as physical activity and nutrition. However, as we included participants prior to switching as the comparator group, it is unlikely that there were large enough shifts in physical activity and nutrition over time to contribute to the change in weight gain before and after TLD transition. We also used complete case analysis to address missing data, which may potentially introduce bias. However, as the amount of missing outcome data was small (3%) and was missing at random, we believe that bias to be minimal.

## 5 CONCLUSIONS

This study provides additional weight data on the TLD transition. While there was an increased risk of developing a high BMI of ≥25 kg/m^2^ for PLWH on TLD compared to PLWH on non‐TLD ART, only about half of those undergoing regimen change gained weight. It will be important to monitor for continued weight gain among men and women beyond the first 2 years after TLD switch, which could predispose to potential downstream metabolic effects. Morbidity linked to DTG will need to be further investigated, particularly in older age groups at risk for multiple NCDs. These potential risks associated with weight gain should be weighed against the demonstrated superiority if INSTI‐based regimens in achieving viral suppression. When considering switching to TLD, the metabolic effects of weight gain after switching to TLD should be weighed against the benefits of a DTG anchor.

## COMPETING INTERESTS

The authors have no competing of interests to disclose.

## AUTHORS' CONTRIBUTIONS

JAA, ALE and DC conceived of the presented research idea. EB, MI, HK, JO, JM and VS carried out the data collection, laboratory activities and reviewed the collected data for quality and reliability. ALE designed the model and analysed the data. NFD verified underlying data. ALE, DC, JAA, TAC, NFD and CSP contributed to the interpretation of the results. ALE and DC took the lead in writing the manuscript. CSP and JAA were in charge of overall direction and planning. All authors provided critical feedback and helped shape the research, analysis and manuscript. All authors approved the final submitted manuscript.

## FUNDING

This work was supported by the President's Emergency Plan for AIDS Relief via a cooperative agreement between the Henry M. Jackson Foundation for the Advancement of Military Medicine, Inc. and the U.S. Department of Defense (W81XWH‐11‐2‐0174 and W81XWH‐18‐2‐0040).

## DISCLAIMER

The views expressed are those of the authors and should not be construed to represent the positions of the US Army or the Department of Defense. The investigators have adhered to the policies for protection of human subjects as prescribed in Army Regulation 70‐25.

## Data Availability

The data sets generated and/or analysed during the current study are not publicly available due to privacy protections but are available from the corresponding author on reasonable request. The Henry M. Jackson Foundation for the Advancement of Military Medicine (HJF) and the Water Reed Army Institute of Research (WRAIR) are committed to safeguarding the privacy of research participants. Distribution of data will require compliance with all applicable regulatory and ethical processes, including establishment and approval of an appropriate data‐sharing agreement. To request a minimal data set, please contact the data coordinating and analysis center (DCAC) at PubRequest@hivresearch.org and indicate the RV329 study along with the name of the manuscript.
